# Supplementary material for: miR-135a Suppresses Granulosa Cell Growth by Targeting Tgfbr1 and Ccnd2 during Folliculogenesis in Mice
Source: Cells. 2021 Aug 17;10(8):2104. doi: 10.3390/cells10082104 (PMC8394614; doi:10.3390/cells10082104)
Supplement: Supplementary file 1 [file cells-10-02104-s001.zip › Figure S1.pdf]

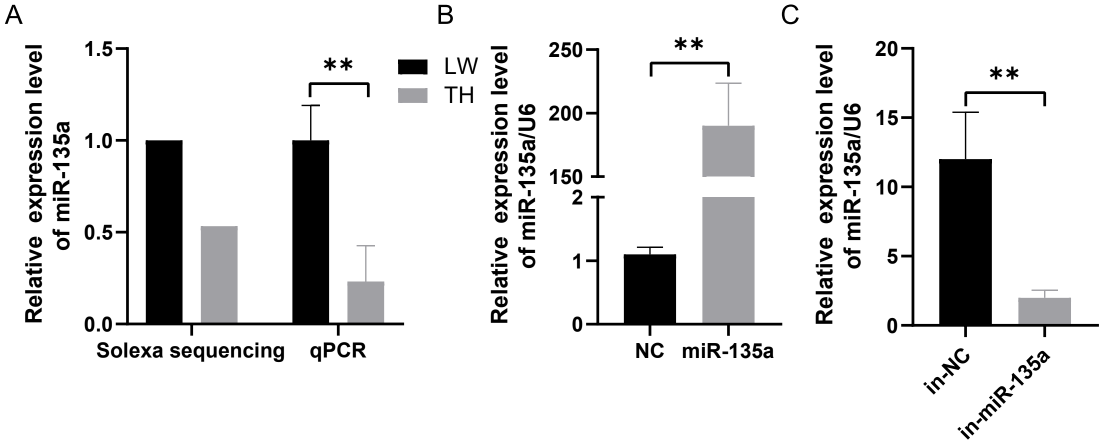

Figure S1. qPCR analysis of miR-135a in porcine follicles and murine GCs. (A) qPCR analysis of miR-135a expression in pre-ovulatory ovarian follicles between Large White (LW) and Chinese Taihu (TH) sows. (B) miR-135a overexpression in mGCs. (C) miR-135a inhibition in mGCs. The means ± SD were calculated from three independent experiments. **P < 0.01.
