# Supplementary material for: miR-135a Suppresses Granulosa Cell Growth by Targeting Tgfbr1 and Ccnd2 during Folliculogenesis in Mice
Source: Cells. 2021 Aug 17;10(8):2104. doi: 10.3390/cells10082104 (PMC8394614; doi:10.3390/cells10082104)
Supplement: Supplementary file 1 [file cells-10-02104-s001.zip › Table S1.pdf]

Table S1. Sequences of primers and oligos (5'-3').

Primer name	Sequences (5'-3')	Application
miR-135a-Loop	CTCAACTGGTGTCGTGGAGTCGGCAATTCAGTTGAGTCACATAG	qPCR
U6-Loop	CAGTTGAGAAAAATATGGAACGCT	
miR-135a-qPCR-F	CTGGTAGGTATGGCTTTTTAT	qPCR
miR-135a-qPCR-R	TCAACTGGTGTCGTGGAG	
U6-F	CTGGTAGGGTGCTCGCTTCGGCAG	qPCR
U6-R	CAACTGGTGTCGTGGAGTCGGC	
Tgfbr1-qPCR-F	TGGGCTTAGTGTTCTGG	qPCR
Tgfbr1-qPCR-R	CTGTTGGCTGAGTTGTGA	
Ccnd2-qPCR-F	GGGAACTGGTAGTGTTGGGTAAG	qPCR
Ccnd2-qPCR-R	AATCATCGACGGCGGGTAC	
Luc-miR-135a-D4-Mut1-F	TAAGCCTGTCTGGTCCTCAGAATCGATCTACCTTGCTTAC	site-directed mutagenesis PCR
Luc-miR-135a-D4-Mut1-R	TAAGACTGAGAAGGAGGTAAGCAAGGTAGATCGATTCTGAGGACC	
Luc-miR-135a-D4-Mut2-F	ACACACACAGGAGCCAGGCTTATGAATCATGGCCCA	site-directed mutagenesis PCR
Luc-miR-135a-D4-Mut2-R	TCTAGCTAGAAGTTGGGCCATGATTCATAAGCCTGGCTCC	
Ccnd2-ChIP-F	TCTGTGCAGGATAACACCGAGAC	ChIP-qPCR
Ccnd2-ChIP-R	CTATCAATGGCAGCGGGAAT	
miR-135a-ChIP-F	TCCACTTCCCTAAGCCTGTC	ChIP-qPCR
miR-135a-ChIP-R	ATGAGCGAGCAATAGAATCAC	
siRNA-NC	UUCUCCGAACGUGUCACGUTT	siRNA
siRNA-Ccnd2	CCAAGCUGAAAGAGACCAUTT	siRNA
siRNA-Tgfbr1	CCAGGACCAUUGUGUUACATT	siRNA
